# Chemsex Interventions for Men Who Have Sex With Men: A Systematic Review and Meta-Analysis

**DOI:** 10.7759/cureus.95731

**Published:** 2025-10-30

**Authors:** Kiriana Lagden

**Affiliations:** 1 Global Public Health, Queen Mary University of London, London, GBR

**Keywords:** chemsex, chemsex interventions, high-risk sexual behaviour, men who have sex with men (msm), sexualised drug use

## Abstract

Behavioural and pharmacological interventions aimed at reducing chemsex practices (the use of psychoactive drugs before or during sexual activity) have been introduced; however, the effectiveness of these interventions remains unclear. This systematic review aims to summarise the effectiveness of bio-behavioural chemsex interventions. PubMed and EMBASE databases were searched, without date and setting restrictions, for studies on chemsex interventions for men who have sex with men (MSM). Eligible studies were assessed, and data were extracted on primary outcomes (high-risk sexual behaviours) and secondary outcomes (sexually transmitted infections (STIs) and human immunodeficiency virus (HIV) incidence, adherence to post-exposure prophylaxis after sexual exposure (PEPSE) and anti-retroviral therapy (ART)). The results were presented quantitatively through a meta-analysis. A total of twelve studies (1,711 participants) were included in the review, while nine studies (1,145 participants) contributed to the meta-analysis. Bio-behavioural chemsex interventions decreased the number of episodes of unprotected anal intercourse (UAI) with serodiscordant partners (MD: -1.30, 95% CI: -1.58 to -1.03; p<0.001). Interventions also decreased the total number of sexual partners and the number of partners where UAI was used, though not significantly. The use of psychoactive substances during sexual activities was not reduced among those who had interventions. Bio-behavioural chemsex interventions reduce the risk of UAI with serodiscordant partners, a high-risk factor for HIV seroconversion. More research is needed on other benefits of chemsex interventions.

## Introduction and background

Chemsex is the use of psychoactive drugs, such as methamphetamine, mephedrone, and gamma-hydroxybutyrate/gamma-butyrolactone (GHB/GBL), before or during sexual activity with the intention of enabling, enhancing, and prolonging sexual interactions [1]. During chemsex, these drugs, taken through a variety of methods (smoking, swallowing, snorting, etc.), are thought to trigger intense feelings of sexual arousal, lower inhibitions, and allow engagement in sex for sustained periods [2]. The practice has become increasingly common among men who have sex with men (MSM) since it emerged in Western Europe, North America, Southeast Asia, and Australia around 2011 [3]. The prevalence of chemsex differs by location and setting, ranging from 3-29% [1,4-6].

Chemsex has been associated with certain risky sexual behaviours. These include unprotected anal intercourse (UAI), sex with multiple partners, and sex with serodiscordant (differing HIV status) partners [1]. Such behaviours increase the risk of transmission of blood-borne viruses (BBVs), such as HIV and hepatitis C virus (HCV), as well as sexually transmitted infections (STIs). MSM are already disproportionately affected by HIV [7,8]; however, some studies have shown that this may further increase if participating in chemsex [5,9], and others have reported a relationship between the practice and an increased risk of STIs [10-13]. Additional harms such as accidental overdose, symptomatic withdrawal effects, and psychosocial changes such as depression, anxiety, psychosis, memory loss, and personality changes can result from chemsex [14].

Since chemsex has been associated with risky sexual behaviours and other physical and psycho-social harms, there have been pharmacological and behavioural interventions designed to target its practice and address its risks and harms. Studies have explored the use of pharmacological agents, such as mirtazapine, naltrexone, benzodiazepines, and barbiturates [15,16], though these treatments are not widely accepted [17,18]. Behavioural interventions, including contingency management [19,20], motivational interviewing (MI) [15], cognitive-based therapy (CBT), and relapse analysis, have also been used [21]. Other interventions include harm reduction strategies, such as the transition to less potent forms of administration of substances [15,22] and the use of pre-exposure prophylaxis (PrEP) or post-exposure prophylaxis following sexual exposure (PEPSE) to reduce the likelihood of HIV infection [23].

There is published literature on a variety of pharmacological and behavioural interventions that can potentially be utilised for MSM who participate in chemsex; however, there is a knowledge gap on the effectiveness of these interventions, which this systematic review aims to fill.

## Review

Methods

Registration and Protocol

The review protocol is registered on PROSPERO (CRD42021238057), and the review is reported in accordance with the Preferred Reporting Items for Systematic Reviews and Meta-Analyses (PRISMA) statement [24]. As a systematic review of published studies, ethical approval was not required.

Search Strategy and Eligibility Criteria

PubMed and EMBASE were searched from inception till 2 March 2021 for published studies assessing chemsex interventions among MSM. Chemsex was defined as sexual activity performed while under the influence of psychoactive drugs, including methamphetamine, mephedrone, and GBL/GHB. Chemsex interventions could be delivered in any setting and were defined as any form of bio-behavioural intervention used with the aim of reducing harms associated with chemsex.

Only published studies in the English language were sought. The search strategy (Tables 1-2) was specified to capture all potentially eligible studies.

**Table 1:** Search terms used for the PubMed database

PubMed: Query	Results
"Men who have sex with men"[Title/Abstract] OR "Homosexual men"[Title/Abstract] OR "Gay men"[Title/Abstract] OR "Gay male"[Title/Abstract] OR "Bisexual men"[Title/Abstract] OR "Bisexual male"[Title/Abstract] OR Sexual and Gender Minorities[MeSH Terms]	25,721
"Illicit drugs"[Title/Abstract] OR "Psychotropic drugs"[Title/Abstract] OR 4-Butyrolactone[Title/Abstract] OR Methamphetamine[Title/Abstract] OR "Sodium Oxybate"[Title/Abstract] AND Sex[Title/Abstract]	1,733
Chemsex[Title/Abstract] OR Sexualised drug use[Title/Abstract]	225
Intervention[All Fields] OR "Sexual health service"[All Fields] OR "Drug service"[All Fields] OR Substance-Related Disorder/therapy[All fields]	8,834,400
("Men who have sex with men"[Title/Abstract] OR "Homosexual men"[Title/Abstract] OR "Gay men"[Title/Abstract] OR "Gay male"[Title/Abstract] OR "Bisexual men"[Title/Abstract] OR "Bisexual male"[Title/Abstract] OR Sexual and Gender Minorities[MeSH Terms]) AND ("Illicit drugs"[Title/Abstract] OR "Psychotropic drugs"[Title/Abstract] OR 4-Butyrolactone[Title/Abstract] OR Methamphetamine[Title/Abstract] OR "Sodium Oxybate"[Title/Abstract] AND Sex[Title/Abstract])	487
("Men who have sex with men"[Title/Abstract] OR "Homosexual men"[Title/Abstract] OR "Gay men"[Title/Abstract] OR "Gay male"[Title/Abstract] OR "Bisexual men"[Title/Abstract] OR "Bisexual male"[Title/Abstract] OR Sexual and Gender Minorities[MeSH Terms]) AND (Chemsex[Title/Abstract] OR Sexualised drug use[Title/Abstract])	175
(("Men who have sex with men"[Title/Abstract] OR "Homosexual men"[Title/Abstract] OR "Gay men"[Title/Abstract] OR "Gay male"[Title/Abstract] OR "Bisexual men"[Title/Abstract] OR "Bisexual male"[Title/Abstract] OR Sexual and Gender Minorities[MeSH Terms]) AND ("Illicit drugs"[Title/Abstract] OR "Psychotropic drugs"[Title/Abstract] OR 4-Butyrolactone[Title/Abstract] OR Methamphetamine[Title/Abstract] OR "Sodium Oxybate"[Title/Abstract] AND Sex[Title/Abstract])) OR (("Men who have sex with men"[Title/Abstract] OR "Homosexual men"[Title/Abstract] OR "Gay men"[Title/Abstract] OR "Gay male"[Title/Abstract] OR "Bisexual men"[Title/Abstract] OR "Bisexual male"[Title/Abstract] OR Sexual and Gender Minorities[MeSH Terms]) AND (Chemsex[Title/Abstract] OR Sexualised drug use[Title/Abstract]))	615
((("Men who have sex with men"[Title/Abstract] OR "Homosexual men"[Title/Abstract] OR "Gay men"[Title/Abstract] OR "Gay male"[Title/Abstract] OR "Bisexual men"[Title/Abstract] OR "Bisexual male"[Title/Abstract] OR Sexual and Gender Minorities[MeSH Terms]) AND ("Illicit drugs"[Title/Abstract] OR "Psychotropic drugs"[Title/Abstract] OR 4-Butyrolactone[Title/Abstract] OR Methamphetamine[Title/Abstract] OR "Sodium Oxybate"[Title/Abstract] AND Sex[Title/Abstract])) OR (("Men who have sex with men"[Title/Abstract] OR "Homosexual men"[Title/Abstract] OR "Gay men"[Title/Abstract] OR "Gay male"[Title/Abstract] OR "Bisexual men"[Title/Abstract] OR "Bisexual male"[Title/Abstract] OR Sexual and Gender Minorities[MeSH Terms]) AND (Chemsex[Title/Abstract] OR Sexualised drug use[Title/Abstract]))) AND (Intervention[All Fields] OR "Sexual health service"[All Fields] OR "Drug service"[All Fields] OR Substance-Related Disorder/therapy[All fields])	443

**Table 2:** Search terms used for the EMBASE database

EMBASE: Query	Results
'men who have sex with men':ab,ti OR 'homosexual male':ab,ti OR 'gay male':ab,ti OR 'gay men':ab,ti OR 'bisexual men':ab,ti OR 'bisexual male':ab,ti OR (sexual AND 'gender minority'/exp)	32,066 (#1)
('illicit drug':ab,ti OR 'psychotropic agent':ab,ti OR 'gamma butyrolactone':ab,ti OR methamphetamine:ab,ti OR 'oxybate sodium':ab,ti) AND sex:ab,ti	2,146 (#2)
chemsex:ab,ti OR 'sexualised drug use':ab,ti	371 (#3)
intervention OR 'sexual health service' OR 'drug service' OR 'drug therapy'	6,714,443 (#4)
('men who have sex with men':ab,ti OR 'homosexual male':ab,ti OR 'gay male':ab,ti OR 'gay men':ab,ti OR 'bisexual men':ab,ti OR 'bisexual male':ab,ti OR (sexual AND 'gender minority'/exp)) AND (('illicit drug':ab,ti OR 'psychotropic agent':ab,ti OR 'gamma butyrolactone':ab,ti OR methamphetamine:ab,ti OR 'oxybate sodium':ab,ti) AND sex:ab,ti)	716 (#5 = #1 AND #2)
('men who have sex with men':ab,ti OR 'homosexual male':ab,ti OR 'gay male':ab,ti OR 'gay men':ab,ti OR 'bisexual men':ab,ti OR 'bisexual male':ab,ti OR (sexual AND 'gender minority'/exp)) AND (chemsex:ab,ti OR 'sexualised drug use':ab,ti)	281 (#6 = #1 AND #3)
('men who have sex with men':ab,ti OR 'homosexual male':ab,ti OR 'gay male':ab,ti OR 'gay men':ab,ti OR 'bisexual men':ab,ti OR 'bisexual male':ab,ti OR (sexual AND 'gender minority'/exp)) AND ('illicit drug':ab,ti OR 'psychotropic agent':ab,ti OR 'gamma butyrolactone':ab,ti OR methamphetamine:ab,ti OR 'oxybate sodium':ab,ti) AND sex:ab,ti OR (('men who have sex with men':ab,ti OR 'homosexual male':ab,ti OR 'gay male':ab,ti OR 'gay men':ab,ti OR 'bisexual men':ab,ti OR 'bisexual male':ab,ti OR (sexual AND 'gender minority'/exp)) AND (chemsex:ab,ti OR 'sexualised drug use':ab,ti))	925 (#7 = #5 OR #6)
		(('men who have sex with men':ab,ti OR 'homosexual male':ab,ti OR 'gay male':ab,ti OR 'gay men':ab,ti OR 'bisexual men':ab,ti OR 'bisexual male':ab,ti OR (sexual AND 'gender minority'/exp)) AND ('illicit drug':ab,ti OR 'psychotropic agent':ab,ti OR 'gamma butyrolactone':ab,ti OR methamphetamine:ab,ti OR 'oxybate sodium':ab,ti) AND sex:ab,ti OR (('men who have sex with men':ab,ti OR 'homosexual male':ab,ti OR 'gay male':ab,ti OR 'gay men':ab,ti OR 'bisexual men':ab,ti OR 'bisexual male':ab,ti OR (sexual AND 'gender minority'/exp)) AND (chemsex:ab,ti OR 'sexualised drug use':ab,ti))) AND ('intervention'/exp OR intervention OR 'sexual health service'/exp OR 'sexual health service' OR 'drug service' OR 'drug therapy'/exp OR 'drug therapy')	295 (#8 = #7 AND #4)

For a study to be included, it had to meet all of the PICO framework [25] inclusion criteria, as demonstrated in Table 3.

**Table 3:** Population, intervention, comparison, and outcomes (PICO) criteria for study inclusion

PICO criteria for study inclusion	
Criteria	Definition
Population	Gay men, bisexual men and men who have sex with men (including those who do not necessarily identify as gay or bisexual) who engage with chemsex and chemsex interventions
Intervention	Bio-behavioural interventions - any form of interventions whether that be through sexual health or drug services, delivered in any setting (including traditional settings, outreach services, community groups etc.)
Comparison	Gay men, bisexual men, men who have sex with men (including those who do not necessarily identify as gay or bisexual) who engage with chemsex but who did not have chemsex bio-behavioural interventions or who had a placebo
Outcomes	Primary outcomes: high-risk sexual behaviours (including unprotected anal intercourse), STI incidence and HIV incidence. Secondary outcomes: additional health outcomes, including onset of and adherence to post-exposure prophylaxis after sexual exposure to HIV (PEPSE) and adherence to anti-retroviral therapy (ART)

This means that a study had to have a population of MSM who engage with chemsex, split into an intervention group who received a bio-behavioural intervention and a control group who received either no intervention or a placebo. The study also had to include an outcome of high-risk sexual behaviours, STI incidence, HIV incidence, or additional health outcomes related to PEPSE or ART use to be eligible.

Screening of the Studies

EndNote software [26] was used to remove duplicate studies, and Rayyan (Rayyan Systems Inc., Cambridge, MA) software [27] was used to manage the data screening process. Titles and abstracts were screened by two independent reviewers to identify studies that potentially met the inclusion criteria. Full texts were then sought and reviewed for potentially eligible articles. In instances where the title and abstract provided insufficient information, full texts were sought and screened to determine eligibility. Where necessary, study authors were contacted for additional information on studies, for their guidance and supervision during the development of this review, and for their assistance with the screening and data extraction of included studies.

Data Extraction

Data extraction was carried out using Microsoft Excel (Microsoft® Corp., Redmond, WA) software. Extracted data from each study included characteristics of the study (authors, title, design, year, country, and sample size), participants (demographics and substance use), chemsex intervention, and outcome assessment.

Assessment of Risk of Bias

Although both randomised and non-randomised studies had been sought, eligible studies were randomised studies. These studies were assessed using the Cochrane Risk of Bias 2 (RoB 2) tool [28].

Statistical Analysis

There were insufficient data to be pooled for most outcomes of interest and follow-up periods. However, meta-analyses were conducted where there was sufficient data and if the participants and interventions were deemed similar enough for pooling to make sense. All data were continuous, analysed as the mean difference (MD) with 95% confidence intervals (CIs). Heterogeneity between studies was quantified using I2, and the thresholds of low (25%), average (50%), and substantial (75%) were used [29]. A fixed effect model was used unless the heterogeneity was significant, in which case a random effects model was used. It was not possible to assess publication bias using funnel plots as there were fewer than 10 studies for meta-analysis, and so the test power would have been too low to distinguish chance from real asymmetry [30]. All analyses were performed using Review Manager (version 5.4; Cochrane Collaboration, London, UK) software using the inverse variance method to weight the studies [31].

Results

Included and Excluded Studies

**Figure 1:**
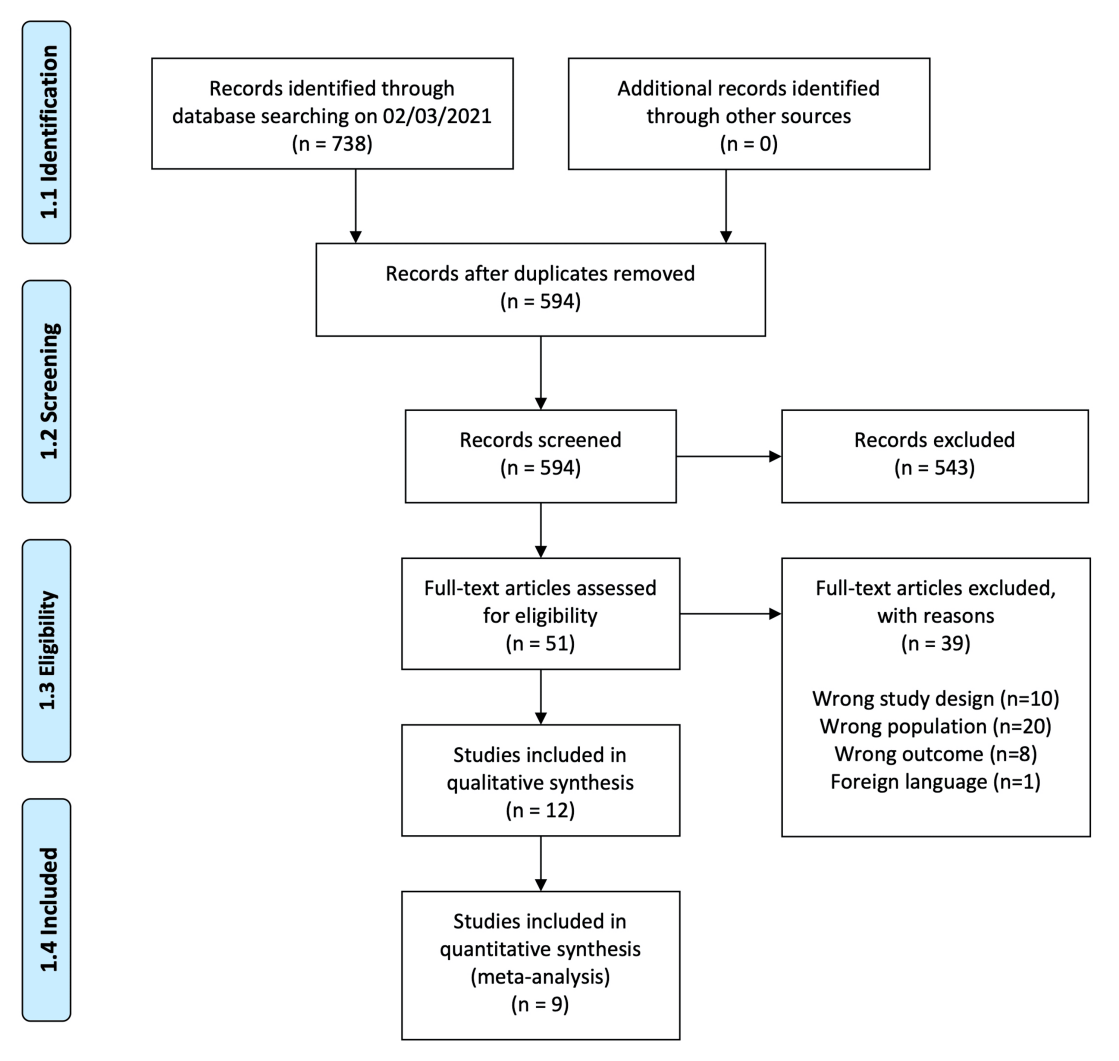
Flowchart of the selection and review process

The systematic search of electronic databases identified 738 articles, 443 from PubMed and 295 from EMBASE. After duplicates were removed, 594 studies were screened by title and abstract, 543 of which were excluded. Full texts for the 51 remaining studies were then screened, and of these, 39 were excluded on the grounds of wrong study design (n=10), wrong population (n=20), wrong outcome (n=8), and foreign language (n=1). The 12 remaining studies (1,711 participants) were included in the systematic review, and nine (1,145 participants) contributed to the meta-analysis. The selection process is shown in the PRISMA flowchart (Figure 1).

Study Characteristics

All 12 studies were randomised controlled trials from the USA. The sample size across the studies ranged from 21 [32] to 341 [33], and the mean age of participants ranged from 33.2 to 45.5 years [34,35]. Seven studies provided data on sexuality, and all had a majority of gay men, with other sexualities identified, including bisexual and other/unknown [20,32-34,36-38]. All studies contained participants of differing ethnicities; however, the majority of participants identified as White in all studies, apart from Parsons et al. [37] and Reback et al. [38].

Intervention Types

Despite the search terms, including the three main chemsex drugs (methamphetamine, mephedrone, and GHB/GBL), 11 of the 12 included studies only focused on methamphetamine use, with 100% of participants in these studies being methamphetamine users. The study that included other drugs found that methamphetamine use was around 10% and that participants also used GHB, ecstasy, alkyl nitrites, and cocaine [35].

Interventions were either pharmacological or behavioural. Four studies used pharmacological interventions, two of which utilised mirtazapine, both giving the same dose daily (identical intervention) [39,40], and two of which utilised naltrexone: one used targeted administration [41] and the other used extended-release [42]. The remaining eight studies used behavioural interventions. Two studies used contingency management (CM), where participants received escalating monetary reinforcement for methamphetamine-free urine samples [20,32]. One study used a text-messaging based intervention [38], while another used expressive writing [34], and four studies used counselling or therapy in their interventions, with one using safer-sex counselling sessions [33], one using behavioural activation sessions [36], one using motivational interviewing (MI) and cognitive-based therapy (CBT) [37], and one using personalised cognitive counselling (PCC) [35]. Study length ranged from two months [41] to 12 months [33,37], and mean retention was 88%.

Risk of Bias Assessment

**Figure 2:**
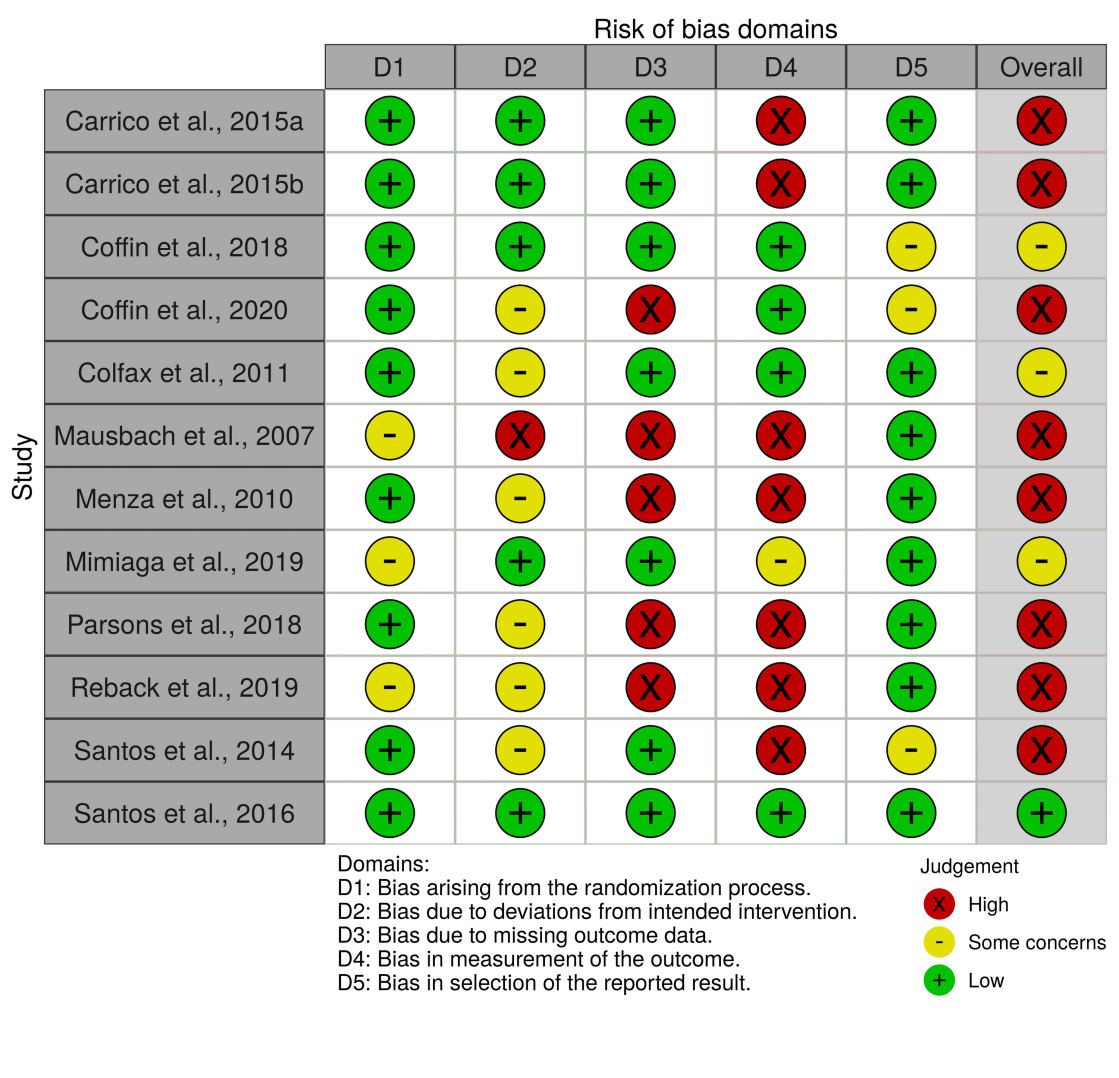
Risk of bias plot Risk of bias plot showing the domain-level judgement for each study [20,32-42].

**Figure 3:**
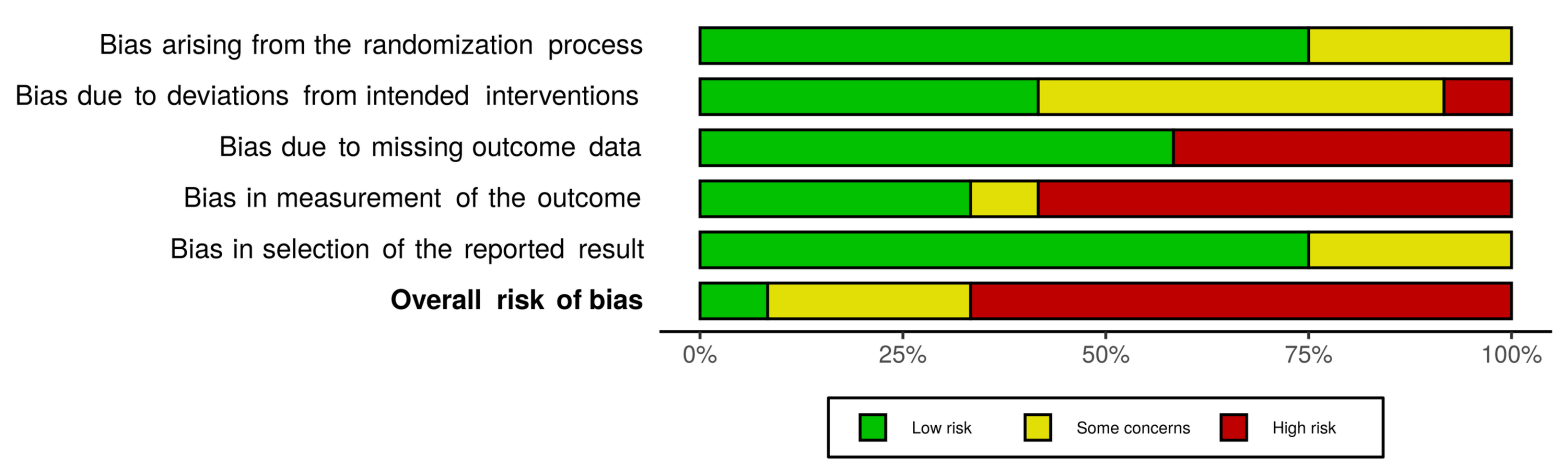
Risk of bias bar chart Risk of bias bar chart showing review author's judgement about each risk of bias domain, presented as percentages across all included studies.

As shown in Figures 2-3, the majority (67%) of studies were rated as having a high risk of overall bias [20,32-35,37-39]. Three studies (25%) were rated as having some concerns for the overall risk of bias [36,40,42], while only one study, Santos et al., was rated as having a low risk of overall bias [41].

Nine studies (75%) reported their method of sequence generation and had a low risk of bias arising from the randomization process [20,32,34,35,37,39-42], while there were some concerns of bias over the remaining three studies, which did not report their randomization process [33,36,38]. In terms of risk of bias due to deviations from intended interventions, a study was considered as having a low risk of bias if there was a relatively high adherence, and this was similar between arms [32,34,36,41,42], some concerns if a study did not report adherence or had low adherence, but again this was similar between arms [20,35,37-40], or high if adherence differed between arms [33]. Five studies (42%) had a high risk of bias due to missing outcome data because they reported high loss to follow-up rates [37-39] or differences in characteristics between those who completed and those who were lost to follow-up [20,33]. When assessing bias in measurement of the outcome, four studies had a low risk of bias as they were double-blind RCTs [39-42]; one had some concerns as although assessors were blinded, participants were aware of their intervention [36]; and seven had a high risk of bias as the participants were aware of the intervention they were receiving and were self-reporting outcomes [20,32-35,37,38]. In complex behavioural intervention studies, it is likely to be difficult to be able to achieve blinding for participants or personnel; however, despite this, these outcomes are still of importance in the context of these interventions. Coffin et al. [39], Coffin et al. [42], and Santos et al. [35] were considered as having some concerns regarding bias in the selection of the reported result, as not all data were presented; for example, means were reported without standard deviations.

Outcomes

Effects of Interventions

Coffin et al. [39], Coffin et al. [42], and Santos et al. [35] met all the inclusion criteria for this systematic review; however, they could not be included in any meta-analyses because of incomplete data [35,39,42]. Coffin et al. [42] used extended-release naltrexone given at four-week intervals for the intervention and placebo for the control, finding that the baseline frequency and subsequent decline were similar between the two, demonstrating no effect of extended-release naltrexone over placebo on sexual risk behaviours [42]. Coffin et al. [39] used mirtazapine as the intervention and matched placebo as the control. It was found that, at three months, changes in sexual risk behaviours were not significant between study arms; however, at six months, those who received mirtazapine had fewer sexual partners (RR: 0.52, 95% CI: 0.27-0.97; p=0.04), fewer episodes of UAI with serodiscordant partners (RR: 0.47, 95% CI: 0.23-0.97; p=0.04), and fewer episodes of receptive UAI with serodiscordant partners (RR: 0.37, 95% CI: 0.14-0.93; p=0.04) than those who received placebo [39]. Santos et al. [35] used PCC as the intervention and rapid HIV testing only as the control, finding that the intervention group had a significant reduction in the number of episodes of UAI while using methamphetamine (RR: 0.26, 95% CI: 0.08-0.84; p=0.02) compared control group [35]. 

Pooled Estimates

Of the eight studies that could be meta-analysed, two of these used pharmacological interventions - mirtazapine [40] and naltrexone [41]. The remaining six studies used behavioural interventions - CM [20], behavioural activation [36], expressive writing [34], text conversations with peer health educators [38], and counselling [32,33]. Table 4 summarises the outcomes of interest measured by the studies.

**Table 4:** Studies contributing to pooled estimates and outcome measured

Studies	Outcome measured
Carrico et al. 2015 [34], Colfax et al. 2011 [40], Santos et al. 2016 [41]	Total number of partners
Carrico et al. 2015 [32], Menza et al. 2010 [20]	Total number of partners UAI
Mausbach et al. 2007 [33], Reback et al. 2019 [38]	Total number of episodes UAI
Carrico et al. 2015, Colfax et al. 2011 [40], Reback et al. 2019 [38]	Total number of partners where substances were used
Colfax et al. 2011 [40], Mimiaga et al. 2019 [36]	Number of episodes UAI with serodiscordant partners
Colfax et al. 2011 [40], Santos et al. 2016 [41]	Number of episodes insertive UAI

**Figure 4:**
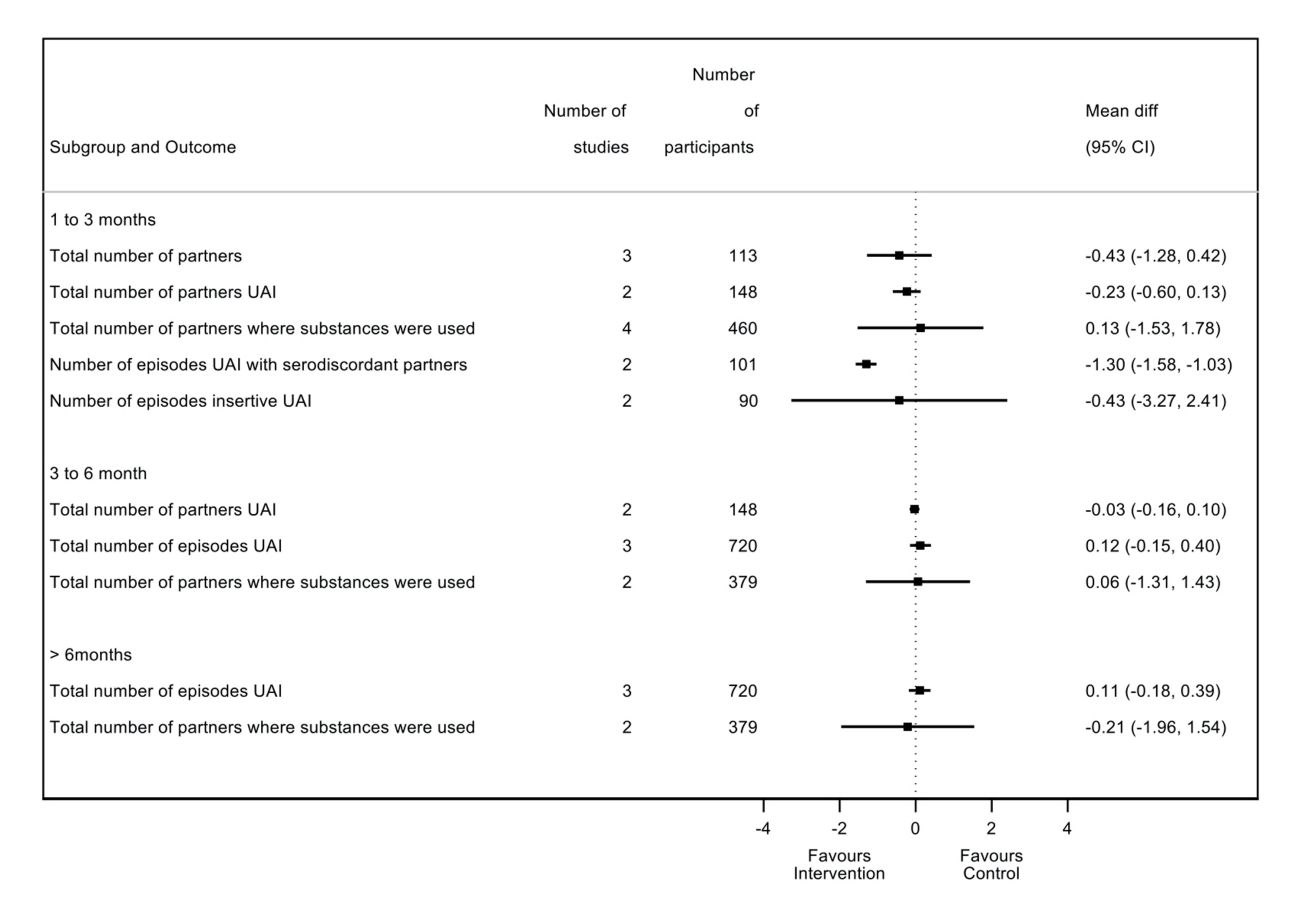
Pooled fixed effect Pooled fixed effect from contributing studies [20,32-34,36,38,40,41].

Pooled fixed effect shows that chemsex intervention reduced the total number of sexual partners after a follow-up at one to three months (MD: -0.43, 95% CI: -1.28 to 0.42; p=0.32; I2=22%) compared with no intervention, though this was not significant (Figure 4). Fixed pooled effect estimate shows that chemsex intervention reduced the number of partners where UAI took place at one to three months (MD: -0.23, 95% CI: -0.60 to 0.13; p=0.21; I2=0%) and at three to six months (MD: -0.03, 95% CI: -0.16 to 0.10; p=0.64; I2=0%). The effect, however, was not statistically significant.

Two studies reported on the relationship between chemsex interventions and the number of episodes of UAI. Reback et al. [38] had multiple trial arms, and as there was no reported cross-over between groups and the outcome data were presented separately, trial arms were treated independently in the meta-analysis [38]. The second study, Mausbach et al. [33], did not assess the number of episodes of UAI at one to three months; therefore, the result could not be pooled [33]. The pooled results from Reback et al. show that the number of episodes of UAI increased in the chemsex intervention group at three to six months (MD: 0.12, 95% CI: -0.15 to 0.40; p=0.38; I2=0%) and at >6 months (MD: 0.11, 95% CI: -0.18 to 0.39; p=0.47; I2=0%) compared to no intervention; however, it was not significant [38].

**Figure 5:**
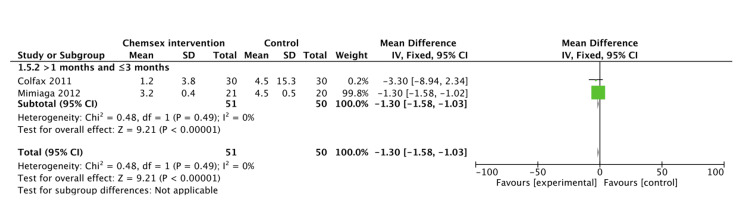
Studies reporting number of episodes of UAI with serodiscordant partners Pooled fixed effect from contributing studies [36,40]. UAI: Unprotected anal intercourse

Pooled random effect estimate shows that the number of partners where substances were used increased in the chemsex intervention group at one to three months (MD: 0.13, 95% CI: -1.53 to 1.78; p=0.88; I2=51%) and at three to six months (MD: 0.06, 95% CI: -1.31 to 1.43; p=0.93; I2=18%), but decreased at >6 months (MD: -0.21, 95% CI: -1.96 to 1.54; p=0.18; I2=45%), compared to no intervention. Nevertheless, none of these results was significant (Figure 5).

**Figure 6:**
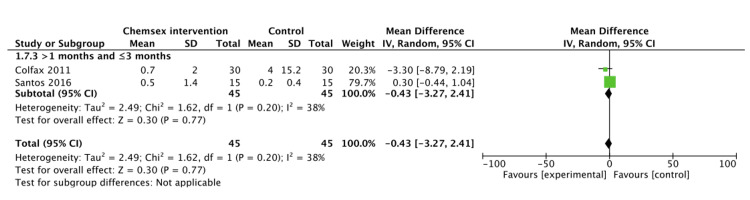
Studies reporting the number of episodes of insertive UAI Pooled random effect from contributing studies [40,41]. UAI: Unprotected anal intercourse

Fixed effect estimate shows that chemsex significantly reduced the number of episodes of UAI with serodiscordant partners at one to three months (MD: -1.30, 95% CI: -1.58 to -1.03; p<0.0001; I2=0%) (Figure 6).

Random effect estimate shows that the number of episodes of insertive UAI decreased in the chemsex intervention group at one to three months (MD: -0.43, 95% CI: -3.27 to 2.41; p=0.77; I2=38%) but was not statistically significant (Figure 6).

Meta-analysis of the secondary outcomes was unable to take place due to the insufficient number of studies. Summaries of findings from studies that reported any of these outcomes are presented in Table 5.

**Table 5:** Findings from studies that reported secondary outcomes

Study	Outcome	Intervention	Sample size	Baseline	1 to 3 months	3 to 6 months	>6 months
Menza et al., 2010 [20]	STI incidence: Gonorrhoea N(%)	Contingency management	70	0 (0)	1 (1.43)		
		Control	57	0 (0)	1 (1.75)		
	STI incidence: Chalmydia N(%)	Contingency management	70	0 (0)	1 (1.43)		
		Control	57	0 (0)	1 (1.75)		
Parsons et al., 2018 [37]	Viral load (mean) ±SD	MI + CBT	107	2.61 (1.44)	2.55 (1.33)	2.35 (1.30)	2.41 (1.33)
		Education control	109	2.62 (1.37)	2.41 (1.27)	2.53 (1.47)	2.45 (1.50)
	CD4 count (mean) ±SD	MI + CBT	107	441.30 (256.48)	451.51 (260.76)	463.60 (259.03)	447.12 (239.54)
		Education control	109	475.28 (268.50)	510.00 (300.62)	507.48 (286.14)	513.38 (316.09)
	ART adherence days (past 14)	MI + CBT	107	9.34 (4.31)	11.27 (4.12)	11.41 (3.89)	11.09 (4.34)
		Education control	109	9.23 (4.21)	12.19 (5.15)	11.59 (3.79)	11.69 (3.83)

Discussion

Summary of the Main Results

This systematic review found that, among MSM, bio-behavioural chemsex interventions decreased the number of episodes of UAI with serodiscordant partners. We found no statistical association between chemsex interventions and the number of partners, number of partners where UAI took place, number of episodes of UAI, or number of sexual episodes where substances were used.

Potential Biases and Limitations in the Review Process

There were several potential biases and limitations in the review process. Despite conducting a comprehensive search of the literature, potentially relevant studies may not have been identified, for example, those that are not in the English language. Although authors were contacted about studies that required additional information, responses were not received, and so three studies were not included in the meta-analysis, as there was insufficient data to do so. There were also inconsistencies in how study outcomes were measured and reported, and so although meta-analysis was possible, some studies were not included in the meta-analysis if they had reported outcomes in an alternative manner.

Pooled effect estimates could be examined for some primary and secondary outcomes because of insufficient studies. Although it was planned to carry out sub-group analysis, it was not possible due to limitations in available data. Another limit of this review is that, despite there being no criteria regarding location in the search strategy and in the inclusion criteria, all studies were conducted in the USA, and so there are concerns regarding the generalisability of these findings to other countries.

Quality and Limitations of the Evidence

Although evidence generated in this review was based on 12 RCTs, the evidence may be biased, with eight of the studies having a high risk of bias and three having some concerns. The main limitations were due to deviations from intended interventions, participants' self-reporting outcomes, and missing outcome data. There are some concerns of bias regarding the finding that interventions decrease the number of episodes of UAI with serodiscordant partners, due to concerns of bias in the studies that contributed to this finding. Colfax et al. [40] had some concerns about bias due to deviations from the intended intervention, as adherence was only 48.5%, while Mimiaga et al. [36] had some concerns arising from bias about the randomisation process and in the measurement of the outcome. For the other outcomes that were not statistically significant, the risk of bias varies. There is a high risk of bias regarding the findings that interventions decrease the total number of partners and total number of partners where UAI took place, as well as for the findings that interventions increased the number of episodes of UAI and the number of partners where substances were used.

In addition to the risk of bias, there are other limitations of the included studies. Most studies had a small sample size, with the maximum number of participants in a study being 341 [33]. A small sample size limits the efficacy of a study and the generalisability of the results to a wider population. Generalisability was also limited in some studies due to restrictions at enrolment, for example, the inclusion of only HIV-negative or HIV-positive MSM [33,34,36]. Furthermore, the long-term efficacy of interventions is unknown, with all study follow-up durations being under 18 months, and some being as short as two months [41].

A limitation of this review is that the search strategy was completed in March 2021; therefore, more recent studies published after this date may not have been captured. Given the evolving nature of chemsex-related research and the ongoing development of new bio-behavioural interventions, future systematic reviews should update the evidence base to include studies published since that time.

Comparison With Previous Findings

There is limited evidence to compare these findings to, as this is an area that has not been extensively studied. Knight et al. [15] conducted a systematic review of interventions for MSM who use methamphetamine and found that some bio-behavioural interventions for methamphetamine use had a statistically significant effect on one or more sexual health-related outcomes, including serodiscordant UAI. The authors were unable to conduct a meta-analysis, and so it is not possible to compare the pooled effect estimate with the review of Knight et al. Despite this, it is clear that there is evidence from this meta-analysis, as well as qualitative evidence from their review, that some bio-behavioural interventions are effective at reducing serodiscordant UAI [15].

Of published studies investigating interventions for methamphetamine use and related sexual risks for MSM, behavioural interventions such as CBT and CM are currently the most researched interventions. Nevertheless, findings have been ambiguous, and there has been limited evidence on their ability to reduce high-risk sexual behaviours [22,43-45]. Similarly, our review did not find that bio-behavioural chemsex interventions are effective at reducing other high-risk sexual behaviours.

Pharmacological interventions could be pooled in subgroup analyses due to data limitations, and so it is not possible to ascertain the impact of specific pharmacological interventions on risky sexual behaviours.

Implication of Findings and Recommendations for Future Research

This review has highlighted that chemsex interventions may help reduce some high-risk sexual behaviours, especially UAI with serodiscordant partners. This is an important finding, as MSM who use methamphetamine are at an increased risk of HIV seroconversion, and those who have UAI with serodiscordant partners are at an even greater risk [46,47].

The benefit of bio-behavioural chemsex interventions over no intervention in reducing the number of partners, number of partners where UAI took place, number of episodes of insertive UAI, number of episodes of UAI, and number of partners where substances were used was unclear and will need further investigations as more data are available.

Further research should be carried out in the rest of the world, particularly in the developed countries, where chemsex has been identified as an increasingly common practice among MSM. Future research into interventions should aim to assess the impact on STI outcomes, HIV outcomes, and adherence to medications such as ART and PEPSE, as evidence regarding these is currently very limited. Finally, this review has highlighted the dearth of research regarding interventions for MSM who use mephedrone and GHB/GBL; therefore, future research should aim to fill this gap.

## Conclusions

This review found that chemsex interventions decreased the number of episodes of UAI with serodiscordant partners, a high-risk sexual behaviour. It found no statistically significant findings relating to the difference between intervention and non-intervention groups regarding the number of partners, number of partners where UAI took place, number of episodes of insertive UAI, number of episodes of UAI, and number of episodes where substances were used for MSM. Future research should focus on the continued development of pharmacological and behavioural interventions for MSM who have chemsex, ensuring that larger sample sizes and standard metrics for data collection are used. Research is also conducted outside of the USA, and there is a focus on gathering data regarding HIV and other STIs, as well as developing interventions for MSM who use mephedrone and GHB/GBL.
